# Evaluation of Novel Enhancer Compounds in Gentamicin-Mediated Readthrough of Nonsense Mutations in Rett Syndrome

**DOI:** 10.3390/ijms241411665

**Published:** 2023-07-19

**Authors:** Keit Men Wong, Eike Wegener, Alireza Baradaran-Heravi, Brenda Huppke, Jutta Gärtner, Peter Huppke

**Affiliations:** 1 Department of Neuropediatrics, Jena University Hospital, 07747 Jena, Germany; brenda.huppke@med.uni-jena.de (B.H.); peter.huppke@med.uni-jena.de (P.H.); 2 Center for Rare Diseases, Jena University Hospital, 07747 Jena, Germany; 3 Department of Pediatrics and Adolescent Medicine, Division of Neuropediatrics, Pediatric Neurology University Medical Center Göttingen, Georg August University Göttingen, 37075 Göttingen, Germany; wegener.eike@gmail.com (E.W.); gaertnj@med.uni-goettingen.de (J.G.); 4 Department of Biochemistry and Molecular Biology, University of British Columbia, Vancouver BC V6T 1Z3, Canada; alireza.baradaran@ubc.ca

**Keywords:** Rett syndrome, readthrough, aminoglycosides, nonsense suppression therapy, methyl-CpG-binding protein

## Abstract

Rett syndrome (RTT), a severe X-linked neurodevelopmental disorder, is primarily caused by mutations in the methyl CpG binding protein 2 gene (*MECP2*). Over 35% RTT patients carry nonsense mutation in *MECP2*, making it a suitable candidate disease for nonsense suppression therapy. In our previous study, gentamicin was found to induce readthrough of *MECP2* nonsense mutations with modest efficiency. Given the recent discovery of readthrough enhancers, CDX compounds, we herein evaluated the potentiation effect of CDX5-1, CDX5-288, and CDX6-180 on gentamicin-mediated readthrough efficiency in transfected HeLa cell lines bearing the four most common *MECP2* nonsense mutations. We showed that all three CDX compounds potentiated gentamicin-mediated readthrough and increased full-length MeCP2 protein levels in cells expressing the R168X, R255X, R270X, and R294X nonsense mutations. Among all three CDX compounds, CDX5-288 was the most potent enhancer and enabled the use of reduced doses of gentamicin, thus mitigating the toxicity. Furthermore, we successfully demonstrated the upregulation of full-length Mecp2 protein expression in fibroblasts derived from *Mecp2^R255X/Y^* mice through combinatorial treatment. Taken together, findings demonstrate the feasibility of this combinatorial approach to nonsense suppression therapy for a subset of RTT patients.

## 1. Introduction

Rett syndrome (RTT) is a complex neurodevelopmental disorder and is the second most prevalent genetic cause of intellectual disability in females [1,2]. Individuals with RTT typically appear asymptomatic during the early postnatal period and undergo developmental regression beginning at 6–18 months, resulting in autonomic dysfunction and loss of acquired motor and speech abilities [3,4]. Approximately 95% of RTT cases are caused by mutations in the X chromosome-linked gene *MECP2*, encoding Methyl-CpG-Binding Protein 2 (MeCP2) [5,6]. A subset of eight common mutations, namely p.R106W, p.R133C, p.T158M, p.R168X, p.R255X, p.R270X, p.R294X, and p.R306C, are responsible for approximately 70% of RTT mutations [7,8]. Notably, approximately 35–40% of RTT-patients carry nonsense mutations, in which a premature termination codon (PTC) arises in the *MECP2* mRNA, leading to the generation of a truncated and nonfunctional MeCP2 protein [7,8]. The transcripts carrying these nonsense mutations activate a process known as nonsense-mediated decay (NMD). NMD is a highly conserved surveillance mechanism that identifies faulty transcripts and marks them for rapid degradation before they can have potentially harmful effects during translation [9]. Due to the high prevalence of nonsense mutations in RTT, inducing ribosomal readthrough of PTCs has emerged as a promising therapeutic approach to restore the production of the full-length protein.

The initial pharmacological chemicals identified to have PTC readthrough activity were the antibiotic aminoglycosides, including G418 and gentamicin [10,11]. These readthrough agents hinder the translation termination at in-frame PTCs by binding to the ribosomal decoding A site, impairing codon/anticodon recognition and enabling pairing of a near-cognate aminoacyl-tRNA (nc-tRNA). The incorporation of an amino acid into the nascent polypeptide chain at the site of PTC allows translation elongation and continuation of full-length protein synthesis [11,12,13,14]. Aminoglycoside-mediated PTC readthrough therapy has been extensively investigated in various rare genetic disorders such as cystic fibrosis (CF) [15], Duchenne muscular dystrophy (DMD) [16,17], Alport syndrome [18], and Epidermolysis Bullosa [19] as well as cancers [20,21,22]. In line with our previous findings, we have demonstrated that aminoglycosides G418 and gentamicin can induce readthrough of PTCs in the RTT-nonsense mutations [23,24]. However, traditional aminoglycosides exhibit only modest readthrough efficacy, and at their effective dose, often lead to ototoxicity and nephrotoxicity [25,26]. Thus, it is important to develop PTC readthrough agents that are both safe and effective for clinical usage.

Many studies have focused on the discovery of more potent readthrough aminoglycosides and optimized their structural properties to minimize associated toxicity [27,28,29,30,31,32]. Among the emerging readthrough agents, the aminoglycoside derivative ELX-02 (also known as NB124) [30] has undergone evaluation in two phase 2 trials in CF patients (NCT04126473 and NCT04135495). While preliminary results indicate limited treatment efficacy, subsequent data assessment has revealed clinically relevant improvement. Nevertheless, a phase 2 trial is underway to investigate the use of ELX-02 in patients with Alport syndrome (NCT05448755). Although many PTC readthrough agents exhibit improved readthrough potency in cellular assay, their activity levels still fall short of the desired range for potential drug candidates. Consequently, researchers have explored alternative strategies to enhance PTC readthrough activity. It has been observed that the aminoglycosides-mediated PTC readthrough can be further enhanced through interactions with other compounds, such as co-treatment with NMD inhibitors [33,34], antimalarial drug mefloquine [18,35], as well as small molecules that target eRF1 or eRF3 [36,37], and TRPC cation channels [38]. Through high-throughput screening, a novel class of phthalimide derivatives (referred to as CDX series compounds) has been identified, which significantly potentiates the readthrough activity of aminoglycosides in human cells [39]. Compound CDX5-1 has been shown to enhance PTC readthrough by G418 in HDQ-P1 cancer cells carrying *TP53* nonsense mutations [39]. Recent in vitro studies have also demonstrated the beneficial effect of CDX5-288 in combination with G418 on *FTLD-GRN* nonsense mutations [40], and the combination of CDX5-288 and CDX6-180 with aminoglycosides on *COL4A5* nonsense mutations [18], thereby demonstrating promising evidence of functionality of the yielded protein. Consequently, combination therapies may enable PTC readthrough at lower doses of aminoglycosides, thereby reducing the associated toxicity.

In our previous investigation, we demonstrated that the aminoglycosides G418 and gentamicin induced readthrough of PTCs in the RTT-nonsense mutations, with a relatively low efficiency of only 10–22% [23]. Therefore, the objective of our current study was to assess whether CDX compounds could enhance the readthrough efficacy, thereby enabling the use of reduced doses of gentamicin in RTT-nonsense mutations. Specifically, we evaluated the effects of CDX5-1, CDX5-288, and CDX6-180 in the HeLa cell lines that harbor the four prevailing *MECP2* nonsense mutations, namely R168X, R255X, R270X, and R294X. We showed that all CDX compounds enhanced the readthrough activity mediated by gentamicin in a dose-dependent manner, with CDX5-288 showing the most remarkable potentiation effect. Notably, we successfully demonstrated the upregulation of full-length Mecp2 protein expression in ex vivo-treated fibroblasts derived from *Mecp2^R255X/Y^* mice through combinatorial treatment. As a result, the CDX compounds offer hope for achieving effective readthrough with lower doses of gentamicin. Our findings contribute proof-of-concept evidence regarding the combinatorial approach for the treatment of RTT patients bearing *MECP2* nonsense mutations.

## 2. Results

### 2.1. CDX Compounds Potentiate Gentamicin-Mediated Readthrough in MECP2 Nonsense Mutations

**Figure 1:**
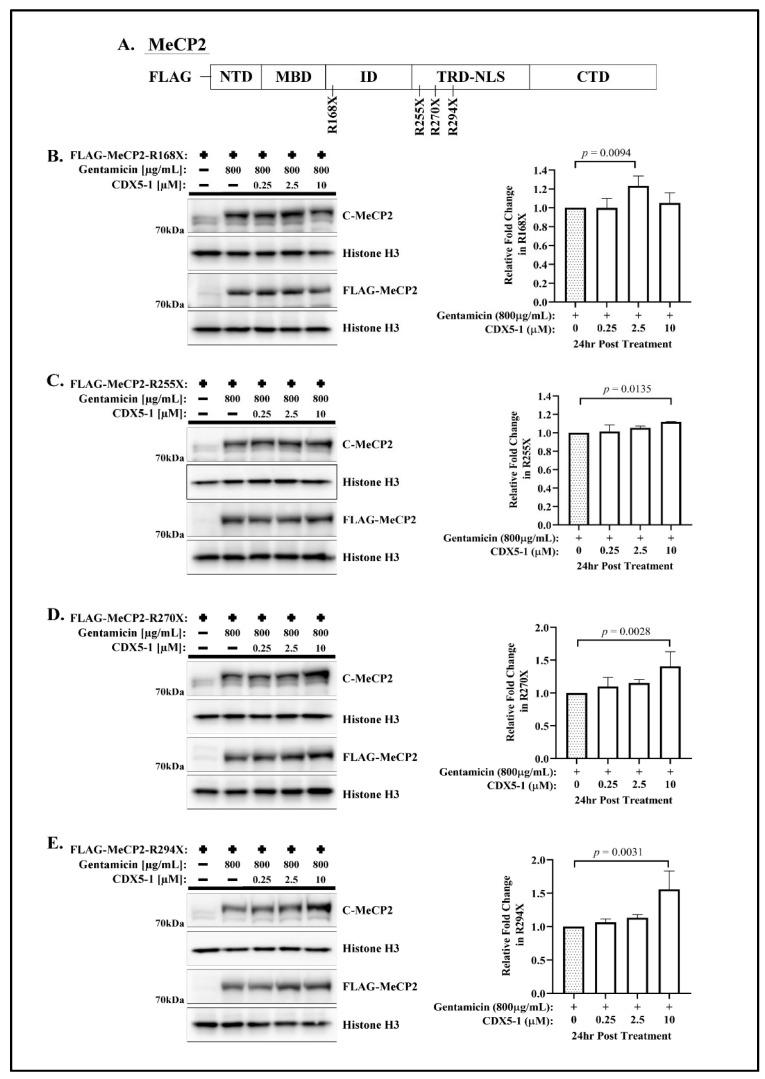
Enhancer CDX5-1 increases the efficiency of gentamicin-mediated readthrough. (**A**) Schematic representation of the FLAG-tagged MeCP2 highlighting the position of the mutants used in this study (NTD, N-terminal domain; MBD, methyl binding domain; ID, internal domain; TRD-NLS, transcription repression domain-nuclear localization signals; CTD, C-terminal domain). Western blot analysis of (**B**) R168X, (**C**) R255X, (**D**) R270X, and (**E**) R290X transfected HeLa cells treated with 800 µg/mL gentamicin and the indicated concentrations of CDX5-1 for 24 h, and probed with the indicated antibodies. The relative fold change of translational readthrough was calculated by comparing the expression of full-length MeCP2 in co-treatment to the expression of full-length MeCP2 in gentamicin treatment alone. Data are given as means ± SD, *n* ≥ 3 independent experiments. Statistical evaluation was performed using one-way ANOVA with Dunnett’s multiple comparison test.

To determine whether CDX compounds could potentiate gentamicin-mediated readthrough, we accessed the readthrough efficacy in the four most common nonsense mutations associated with RTT. In all cases, the mutations introduced an in-frame ochre (UGA) stop codon in place of arginine residues 168, 255, 270, and 294 of MeCP2. Transiently transfected HeLa cells expressing a human *MECP2* cDNA, carrying the R168X (UGAG), R255X (UGAA), R270X (UGAA), and R294X (UGAU) mutations, were subjected to treatment with either gentamicin alone or a combination of CDX compounds and gentamicin for a duration of 24 h. The expression of a full-length MeCP2 was detected using an anti-FLAG and an anti-MeCP2 antibody targeting C-terminal epitope of the protein. The relative fold change in translational readthrough was determined by comparing the expression levels of full-length MeCP2 between the co-treatment groups and the group treated with gentamicin alone. Western blot analysis confirmed the absence of the full-length FLAG-MeCP2 in all untreated cells expressing mutated MeCP2 fusion protein (Figure 1 and Figure S1).

Combining CDX5-1 with gentamicin resulted in a significant increase in the amount of full-length MeCP2 protein when compared to gentamicin treatment alone (Figure 1). CDX5-1 did not exhibit an obvious dose-dependent potentiation effect on gentamicin-mediated readthrough (Figure 1B–E). Quantitative analysis of the full-length MeCP2 protein within the tested range of concentration (up to 10 µM of CDX5-1) indicated that co-treatment with CDX5-1 and gentamicin led to a modest increase in readthrough efficiency in R168X (1.23-fold) (Figure 1B), R255X (1.12-fold) (Figure 1C), R270X (1.40-fold) (Figure 1D) and R294X (1.56-fold) (Figure 1E) compared to cells treated with gentamicin alone. Furthermore, the co-treatment did not result in any noticeable alteration in the expression of truncated FLAG-MeCP2 isoforms across all cell lines carrying one of the four MECP2-nonsense mutations (Figure S1).

**Figure 2:**
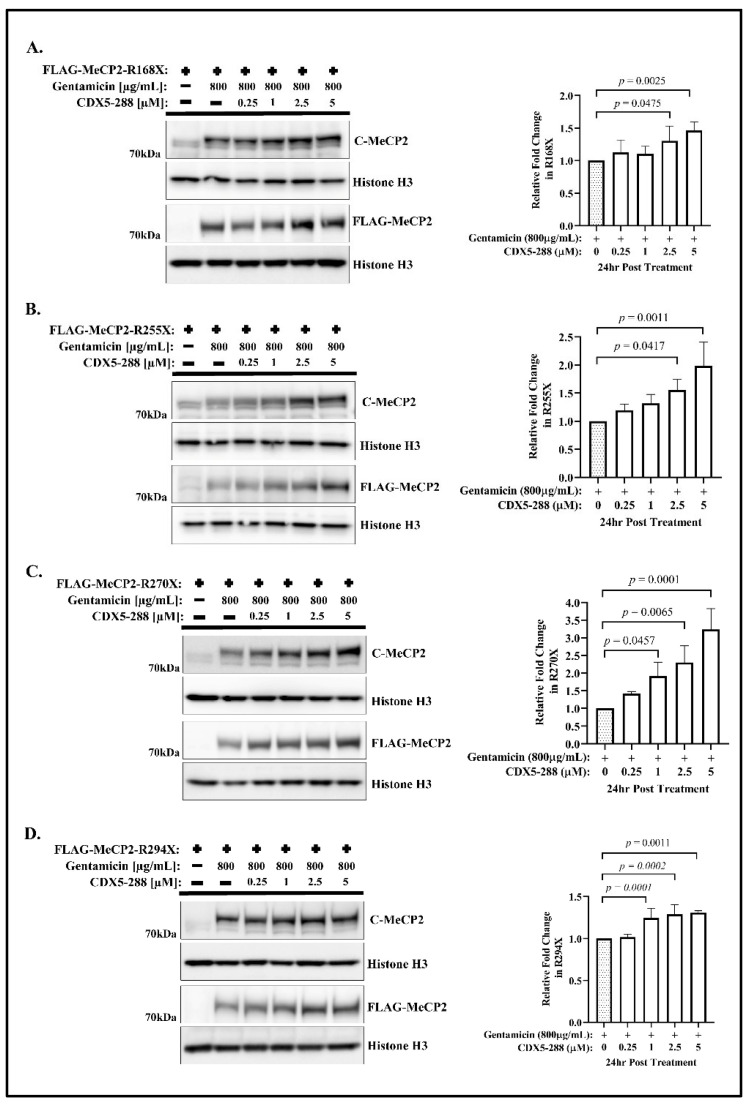
Enhancer CDX5-288 increases the efficiency of gentamicin-mediated readthrough. Western blot analysis of (**A**) R168X, (**B**) R255X, (**C**) R270X, and (**D**) R290X transfected HeLa cells treated with 800 µg/mL gentamicin and the indicated concentrations of CDX5-288 for 24 h, and probed with the indicated antibodies. The relative fold change of translational readthrough was calculated by comparing the expression of full-length MeCP2 in co-treatment to the expression of full-length MeCP2 in gentamicin treatment alone. Data are given as means ± SD, *n* ≥ 3 independent experiments. Statistical evaluation was performed using one-way ANOVA with Dunnett’s multiple comparison test.

Using the same experimental conditions, we assayed the synthesis of the full-length MeCP2 resulting from the combined treatment of CDX5-288 and gentamicin. In contrast to CDX5-1, the co-treatment of CDX5-288 and gentamicin resulted in a dose-dependent increase in readthrough efficiency, as shown in Figure 2. At a concentration of 5 µM of CDX5-288, we observed a significant 1.47-fold increase in R168X (Figure 2A), 2-fold increase in R255X (Figure 2B), 3.23-fold increase in R270X (Figure 2C), and 1.31-fold increase in R294X (Figure 2D). No noticeable alteration in the expression of truncated FLAG-MeCP2 isoforms was observed in any cell line harboring one of the four MECP2-nonsense mutations following the combined treatment (Figure S2).

**Figure 3:**
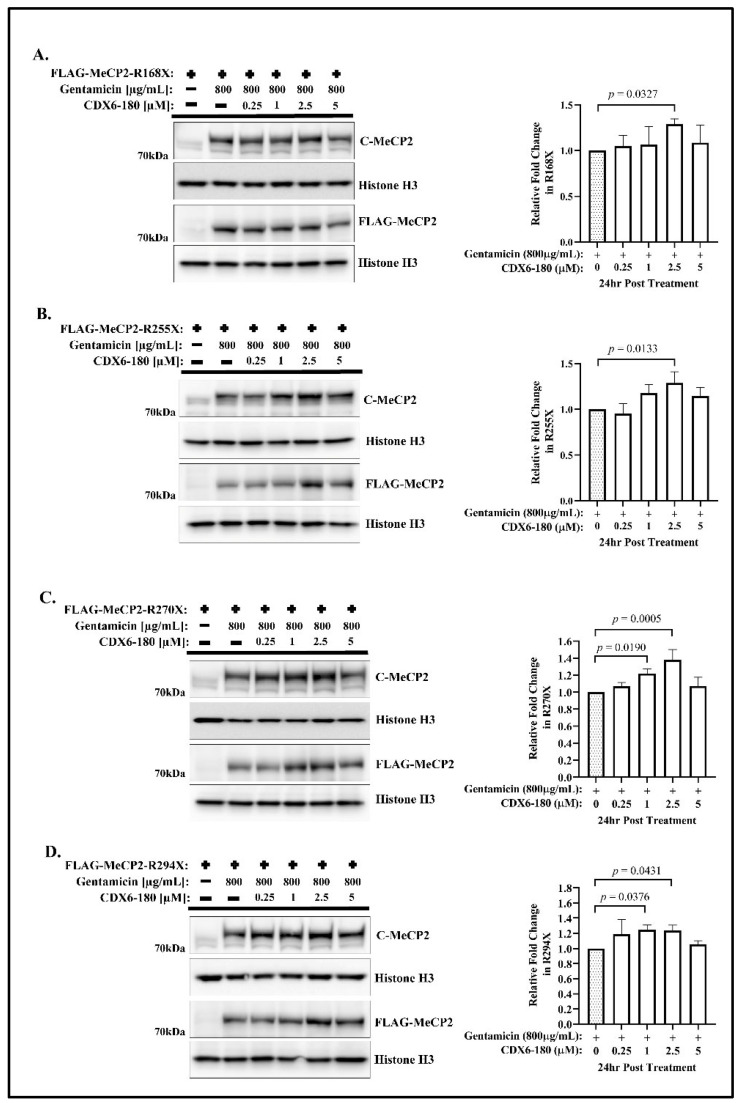
Enhancer CDX6-180 increases the efficiency of gentamicin-mediated readthrough. Western blot analysis of (**A**) R168X, (**B**) R255X, (**C**) R270X, and (**D**) R290X transfected HeLa cells treated with 800 µg/mL gentamicin and the indicated concentrations of CDX6-180 for 24 h, and probed with the indicated antibodies. The relative fold change in translational readthrough was calculated by comparing the expression of full-length MeCP2 in co-treatment to the expression of full-length MeCP2 in gentamicin treatment alone. Data are given as means ± SD, *n* ≥ 3 independent experiments. Statistical evaluation was performed using one-way ANOVA with Dunnett’s multiple comparison test.

Next, we assayed the production of the full-length MeCP2 following the co-treatment of CDX6-180 and gentamicin. Within the tested concentration range, we observed a peak in readthrough efficiency at 2.5 µM, followed by a tendency of decreased readthrough efficiency at 5 µM (Figure 3). At a concentration of 2.5 µM of CDX6-180, the readthrough efficiency exhibited an approximately 1.3-fold increase in both R168X and R255X (Figure 3A,B), 1.38-fold increase in R270X (Figure 3C), and 1.24-fold increase in R294X (Figure 3D). Overall, compared to CDX5-1 and CDX5-288, CDX6-180 showed a narrower therapeutic window in all four mutations. Similarly, the co-treatment did not result in any noticeable alteration in the expression of truncated FLAG-MeCP2 isoforms in any of the cell lines harboring one of the four MECP2-nonsense mutations (Figure S3).

### 2.2. Co-Treatment of CDX Compounds with Gentamicin Did Not Lead to Cytotoxicity

**Figure 4:**
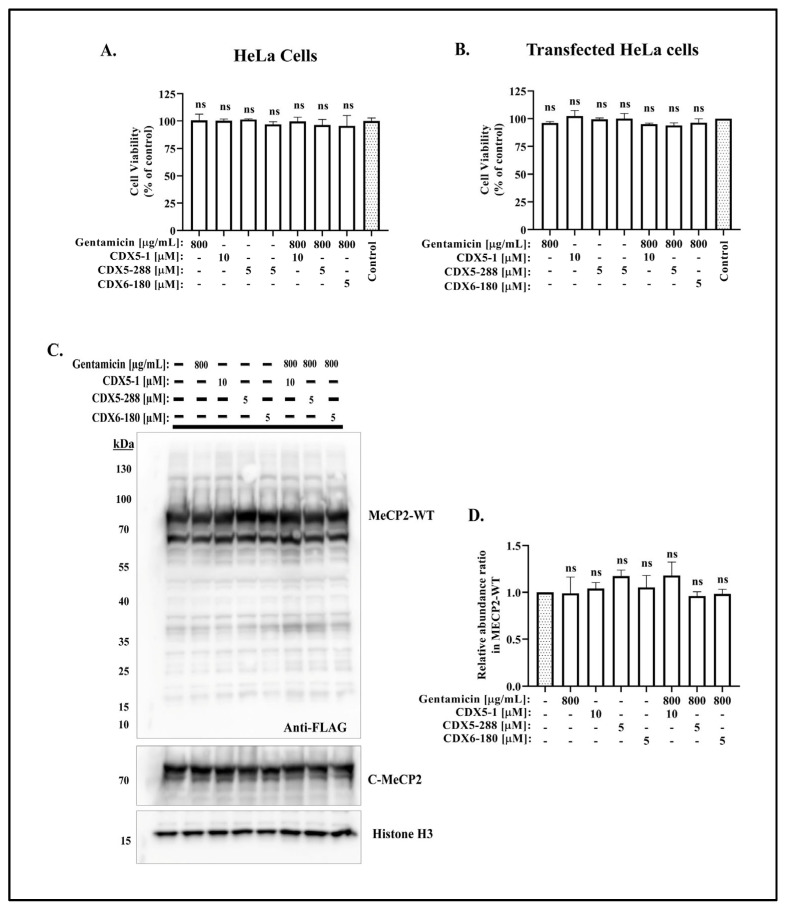
Co-treatment of CDX compounds with gentamicin did not lead to cytotoxicity. Cell viability data for (**A**) HeLa cells and (**B**) FLAG-MeCP2-WT transfected HeLa cells treated with DMSO (control) or indicated concentration of gentamicin and CDX compounds for 24 h. Cell viability was calculated as % of control, and the effect of drug treatment was compared to control. (**C**) Western blot analysis of FLAG-MeCP2-WT transfected HeLa cells treated with indicated concentrations of gentamicin and CDX compounds for 24 h, and probed with the indicated antibodies. (**D**) Quantitative analysis of MeCP2 protein expression after 24 h of treatment. The relative abundance ratio was calculated by comparing the expression of MeCP2 protein in treated cells to the expression of MeCP2 protein in untreated cells, using FLAG densitometric readings. Data are given as means ± SD, *n* = 3 independent experiments. Statistical evaluation was performed using one-way ANOVA with Dunnett’s multiple comparison test. Note that not significant (ns) changes in all samples.

To rule out the potential toxicity of the combination treatment of CDX compounds and gentamicin, we assessed the cell viability using a Cell Counting Kit 8 (WST-8) assay. Our data showed that the co-treatment is not toxic to the cells at the tested condition (Figure 4A,B). Additionally, the wild-type *MECP2* cDNA transfected HeLa cells were treated with various CDX compounds with or without gentamicin for a duration of 24 h, and the expression of wild-type *MeCP2* protein was analyzed through Western blot. Our results showed that the co-treatment of CDX compounds and gentamicin did not induce any significant changes in protein expression as compared to the untreated samples (Figure 4C,D). Taken together, these results indicate that the CDX compounds potentiate gentamicin-mediated readthrough to varying degrees in *MECP2* nonsense mutations, without exhibiting noticeable toxicity.

### 2.3. Readthrough Product Colocalized to Nucleus after Co-Treatment Therapy

**Figure 5:**
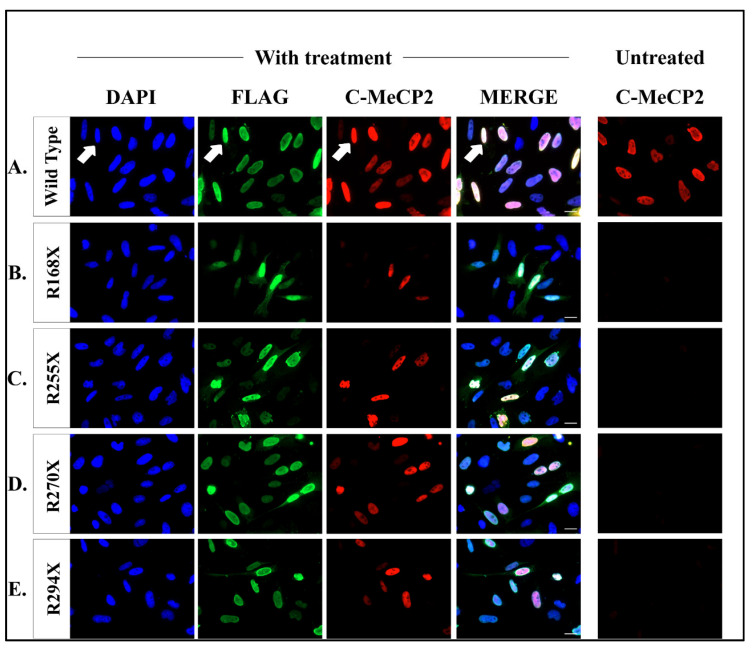
Nuclear localization of restored full-length MeCP2 protein. (**A**) Immunofluorescence studies of wild type-MeCP2, (**B**) R168X, (**C**) R255X, (**D**) R270X, and (**E**) R290X transfected HeLa cells treated with 800 µg/mL gentamicin and 5 µM of CDX5-288 or untreated. Staining of the Anti-FLAG (green signal) and Anti-C-MeCP2 (red signal) correspond with the DAPI nuclei staining (blue signal), thus indicating its nuclear localization (arrow). Scale bars: 20 µm.

To investigate the subcellular localization of the full-length MeCP2 proteins resulting from readthrough, we conducted immunofluorescent cell staining using an antibody that specifically recognized the MeCP2 C-terminus and an anti-FLAG M2 antibody. The HeLa cells were transfected with eukaryotic expression vectors carrying both the wild-type *MECP2* cDNA and mutated *MECP2* cDNAs N-terminally fused to a FLAG-tag; therefore, the colocalization of both antibodies indicated full-length proteins generated from the readthrough (arrow, Figure 5A). To validate this concept, we treated the cells with the most potent compound, CDX5-288, together with gentamicin for all four nonsense mutations. No signal corresponding to C-MeCP2 was detected in untreated cells expressing mutated MeCP2 isoforms (Figure S4). After co-treatment of CDX5-288 and gentamicin, the full-length MeCP2 proteins are localized in the nucleus, similar to the wild-type protein (Figure 5A–E), indicating that the readthrough protein is correctly transported into the nucleus.

### 2.4. CDX5-288 Allows the Use of Reduced Doses of Gentamicin

**Figure 6:**
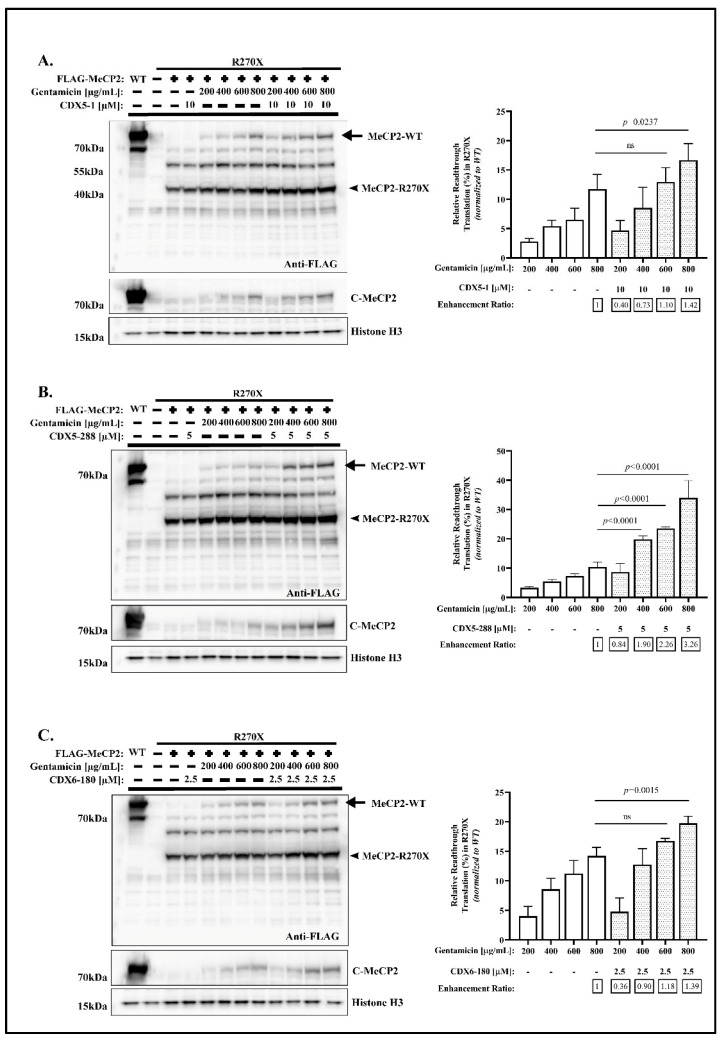
CDX5-288 allows the use of reduced doses of gentamicin. Western blot analysis of R270X transfected HeLa cells that are treated with indicated concentration of gentamicin with or without (**A**) CDX5-1, (**B**) CDX5-288, (**C**) CDX6-180 for 24 h and probed with indicated antibodies. Right: quantitative analysis of full-length MeCP2 protein expression after 24 h of treatment. The relative readthrough translation (%) was calculated by comparing the expression of full-length MeCP2 in the treated samples to the expression of wild-type MeCP2, using the FLAG densitometric readings. The enhancement ratio represents the expression of full-length MeCP2 in cells co-treated with CDX compound and gentamicin relative to the expression of full-length MeCP2 in cells only treated with 800 µg/mL gentamicin alone. Data are given as means ± SD, *n* ≥ 3 independent experiments. Statistical evaluation was performed using one-way ANOVA with Dunnett’s multiple comparison test.

Next, we investigated whether these CDX compounds allow the use of reduced doses of gentamicin and thereby reduced toxicity. Among all four nonsense mutations, R270X displayed the most significant enhancement effect; hence, R270X was chosen for further investigations. For the quantitative analysis, the relative readthrough translation (%) was calculated by comparing the expression of full-length MeCP2 in the treated samples to the expression of wild-type MeCP2. The enhancement ratio represents the expression of full-length MeCP2 in cells co-treated with CDX compound and gentamicin relative to the expression of full-length MeCP2 in cells only treated with 800 µg/mL gentamicin alone. In accordance with previous findings [39], none of the CDX compounds induced PTC readthrough activity when used as a single agent (Figure 6A–C). Untreated R270X-transfected cells did not show any full-length MeCP2 protein, while treatment with gentamicin induced PTC readthrough activity in a dose-dependent manner (Figure 6A–C), consistent with previous findings [23]. In tested concentrations, only the combination of 800 µg/mL of gentamicin and 10 µM of CDX5-1 exhibited a significant 1.42-fold increase in readthrough compared to treatment with 800 µg/mL of gentamicin alone (Figure 6A). This finding suggests that CDX5-1 is unable to facilitate the use of reduced doses of gentamicin. Conversely, when combined with 800 µg/mL of gentamicin, 5 µM of CDX5-288 resulted in a substantial 3.26-fold increase in readthrough (Figure 6B). It is noteworthy that this combinatorial treatment achieved approximately 34% recovery of the full-length MeCP2 protein compared to the wild-type protein (Figure 6B). Importantly, 5 µM of CDX5-288 significantly enhanced the readthrough by 1.9-fold in combination with 400 µg/mL of gentamicin and 2.26-fold in combination with 600 µg/mL of gentamicin compared to treatment with 800 µg/mL of gentamicin alone (Figure 6B). This suggests that CDX5-288 may enable the use of lower doses of gentamicin while maintaining effective readthrough. Similar to the CDX5-1, co-treatment of CDX6-180 and 800 µg/mL of gentamicin was necessary to induce a significant 1.39-fold increase in readthrough (Figure 6C), indicating that CDX6-180 is unable to facilitate the use of reduced doses of gentamicin as well. Collectively, these results suggest that CDX5-288 is the strongest potentiator of gentamicin-mediated readthrough.

### 2.5. Co-Treatment Enhances Readthrough in *Mecp2*^R255X/Y^ Mouse Fibroblasts 

**Figure 7:**
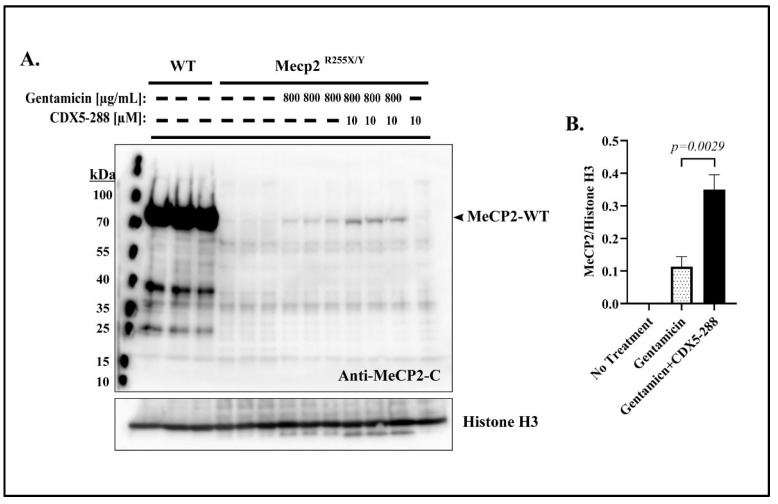
Combinatorial treatment enhances MeCP2 protein expression in *Mecp2*^R255X/Y^ mouse-derived fibroblasts. (**A**) Western blot demonstrating no full-length MeCP2 protein from fibroblasts derived from *Mecp2* ^R255X/Y^ mice, but full-length MeCP2 protein after 4 d treatment with 800 µg/mL gentamicin alone or in combination with CDX5-288. Each lane corresponds to an individual treatment experiment. (**B**) Quantitative analysis of MeCP2 protein expression in *Mecp2*^R255X/Y^ mouse-derived fibroblasts. Data are given as means ± SD. Statistical evaluation was performed using unpaired Welch’s *t*-test.

To further characterize the potentiation effect of CDX5-288, we investigated the readthrough efficiency using fibroblasts derived from *Mecp2*^R255X/Y^ mice. As expected, *Mecp2*^R255X/Y^-derived fibroblasts did not exhibit any expression of full-length Mecp2 protein (Figure 7A). When exposed to a 4-day incubation with 800 µg/mL of gentamicin, *Mecp2*^R255X/Y^ -derived fibroblasts showed the expression of full-length Mecp2 protein (Figure 7A). Remarkably, the co-treatment of gentamicin and CDX5-288 significantly enhanced the expression of full-length Mecp2, achieving a threefold increase compared to gentamicin treatment alone (Figure 7B).

## 3. Discussion

Extensive research efforts are being dedicated to exploring the field of readthrough biology due to its significant potential in addressing the approximate 11% of genetic disorders attributed to nonsense mutations. In particular, aminoglycoside-mediated readthrough is a very promising treatment approach for genetic diseases and cancers caused by PTCs, but widespread clinical application has been severely limited by the high toxicity and low potency of conventional aminoglycosides, prompting the search for superior compounds [11,12,13]. Recently, a new class of compounds known as CDX compounds have been discovered that significantly enhance the readthrough effects of aminoglycosides, leading to increased expression of full-length proteins [18,39,40]. These CDX compounds have generated considerable interest as they offer the potential to improve the therapeutic benefits of aminoglycosides while minimizing adverse effects through reduced dosing. Therefore, the goal of this study was to evaluate the potentiation effect of CDX5-1, CDX5-288, and CDX6-180 on gentamicin-mediated PTC readthrough efficiency in the four most common RTT nonsense mutations, R168X, R255X, R270X, and R294X.

Our study provides evidence for the significant potentiation effect of CDX compounds on gentamicin-mediated nonsense suppression in RTT, without exhibiting noticeable toxicity or affecting the expression of normal MeCP2 protein. Among the three CDX compounds, CDX5-288 emerged as the most potent enhancer of gentamicin-mediated readthrough. This finding aligns with other studies that have also observed the superior readthrough activity of CDX5-288 [18,40]. CDX5-288 demonstrated a dose-dependent potentiation of gentamicin-mediated readthrough in all four nonsense mutations, with the strongest effect observed in the R270X. In a previous study, we showed that gentamicin treatment alone led to PTC readthrough with an efficiency ranging from 10 to 22% depending on the nucleotide context of the nonsense mutations [23]. For instance, in the case of R270X, gentamicin in a concentration of 1 mg/mL resulted in an approximately 12% increase in readthrough efficiency [23]. Co-treatment of 5 µM of CDX5-288 and 800 µg/mL of gentamicin in R270X resulted in the recovery of approximately 34% recovery of the full-length MeCP2 protein compared to the wild-type protein. Even when the concentration of gentamicin was halved (400 µg/mL), CDX5-288 still significantly enhanced readthrough by 1.9-fold compared to treatment with 800 µg/mL of gentamicin alone, highlighting the potent enhancement capability of CDX5-288. Although to a lesser extent, CDX5-1 also showed a modest potentiation effect in all four nonsense mutations. On the other hand, results were less promising for the third compound, CDX6-180. Within the tested concentration range, a peak in readthrough efficiency was observed at 2.5 µM, followed by a subsequent decrease in readthrough efficiency at 5 µM in all four mutations, suggesting a narrow therapeutic window for this particular compound. In our experimental conditions, neither CDX5-1 nor CDX6-180 showed the feasibility of reducing the doses of gentamicin. 

The readthrough efficiency of stop codons is influenced by several factors including the specific sequence of the stop codon as well as the surrounding sequence context upstream and downstream of the stop codon [41,42,43], mRNA stability and availability [44,45], tRNA abundance [46,47], post-translational modification of ribosomal proteins [48], translational initiation factors [49] and release factors [50] as well as metabolic stress [51]. In particular, the identity of the nucleotide at the +4 position immediately downstream of the PTC has been shown to have a significant impact on the efficiency of translational readthrough [52,53]. The four nonsense mutations in this study replace a codon for arginine with an in-frame UGA stop codon. Studies of the UGA stop codon have shown that aminoglycoside-mediated readthrough is highest in the presence of cytosine in the +4 position with some reporting a descending order of influence of C > A = G > U [54,55] and others C > U > A > G [56]. In our previous study of nonsense mutation suppression in RTT, gentamicin-mediated readthrough activity corresponded to the tetranucleotide sequence R294X (UGAU) > R255X (UGAA) > R270X (UGAA) > R168X (UGAG), supporting the latter fourth base order [23]. The addition of CDX compound appeared to interfere with the gentamicin-mediated readthrough activity, for instance, with CDX5-288 showing enhancement in the order of R270X (UGAA) > R255X (UGAA) > R168X (UGAG) > R294X (UGAU). The precise molecular mechanisms underlying the potentiation effect of CDX compounds remain unknown. It is unclear whether they directly or indirectly interact with the ribosome or ribosome-associated proteins, or alter tRNA pairing rates. The combinatorial treatment in the *MECP2*-WT cell line suggests that CDX compounds have no effect on protein synthesis. To our knowledge, CDX compounds do not stimulate gene expression and have recently been reported to have no impact on ribosome biogenesis [39,57]. Further research is needed to elucidate the underlying molecular mechanisms of enhancement.

Aminoglycosides bypass PTC by promoting the incorporation of nc-tRNAs into the ribosomal-A site during protein translation, resulting in a full-length protein with an incorrect amino acid at the PTC site [11]. Therefore, it is crucial to determine the functionality of the rescued protein. While we have demonstrated successful translocation of the full-length MeCP2 protein into the nucleus, functional assays to assess its activity are still lacking in this study. Apart from direct drug injection to the RTT mouse models carrying a nonsense mutation, there is no available functional cell-based assay. To date, it has been shown that G418 restored full-length Mecp2 in *Mecp2*^R294X/Y^ mice in vivo through intracranial ventricular injection [58], while other studies have assessed the readthrough efficiency using ex vivo treatment of patient-derived fibroblasts [29] or *Mecp2* RTT mouse-derived cells [24,59]. Accordingly, we treated fibroblasts derived from *Mecp2*^R255X/Y^ mice with a combination of CDX5-288 and gentamicin for a duration of 4 days, resulting in a successful upregulation of the full-length Mecp2 protein expression.

From a therapeutic standpoint, the dosage of gentamicin employed in this study exceeds the levels typically used in clinical application, where the therapeutic concentrations in pediatric patients generally range from 2 to 9.5 mg/kg/dose. Nevertheless, considering the potentiation effect of CDX5-288, it is plausible to achieve effective and safe therapeutic doses of gentamicin by further increasing the concentration of CDX5-288. Alternatively, significant progress has been achieved in the development of synthetic aminoglycoside derivatives (i.e., NB124/EXL-02), surpassing the performance of traditional aminoglycosides. Importantly, in a mouse model of the lysosomal storage disease mucopolysaccharidosis type-1 Hurler, NB84 has shown noticeable PTC suppression activity and the potential to cross the blood–brain barrier (BBB), leading to the attenuation of central nervous system disease progression [60]. Moreover, unlike aminoglycosides, these NB compounds do not induce the readthrough at nature termination codons (NTCs), making them ideal for selectively targeting PTCs while maintaining high specificity [43,61]. In light of these findings, it would be highly valuable to assess the synergistic effect of CDX compounds in combination with these NB compounds in both in vitro and in vivo RTT mouse models.

Although our results are promising, many questions remain open and have to be addressed in future studies. Firstly, the toxicity of the compounds, especially regarding long-term use, has to be studied in animal models. These experiments will also give an idea about the in vivo efficacy of the compounds, especially if they are performed in already available mouse models carrying different nonsense mutations. Secondly, as it is unknown so far how much MeCP2 is needed to see a clinically relevant effect, we are unable to predict if the readthrough seen in our experiments is sufficient. Unfortunately, such questions will have to wait for a clinical study in humans as the animal models of Rett syndrome have a very limited correlation with the human phenotype. 

In summary, this study adopted an experimental approach, with a primary focus on assessing the potentiating effect of CDX compounds on gentamicin-mediated readthrough in *MECP2* nonsense mutations. Among the CDX compounds tested, CDX5-288 appears the most promising candidate for optimizing aminoglycoside-mediated nonsense suppression. We have provided a ‘proof of principle’ that the combinatorial approach could be beneficial for a subset of RTT patients. Further studies will include more complex validation experiments, such as using animal models with nonsense mutation to assess BBB permeability and the recovery of RTT symptoms. 

## 4. Materials and Methods 

### 4.1. Cell Culture

HeLa cells and mouse fibroblasts were maintained as monolayer cultures growing in Dulbecco’s modified Eagle’s medium (DMEM/low glucose, Gibco, Billings, Montana, MT, USA) supplemented with 10% fetal bovine serum (Biowest, Nuaillé, France), 2 mM L-glutamine (Gibco). Cells were incubated at 37 °C in an atmosphere of 5% CO_2_.

### 4.2. Gentamicin and CDX-Compounds

The aminoglycoside gentamicin was purchased from ratiopharm^®^, Ulm, Germany. All CDX compounds (CDX5-1, CDX5-288 and CDX6-180) were synthesized by the group of Prof. Michel Roberge from the University of British Columbia. The screening assay and structures of the CDX compounds have been described previously [39,40,57].

### 4.3. Transfection and Drug Treatment

HeLa cells were transfected with the eukaryotic expression vectors as described previously [23] using Effectene reagent (QIAGEN, Hilden, Germany) following the manufacturer’s protocol. Briefly, a total of 3 × 10^5^ HeLa cells were plated onto six-well culture plates and grown overnight. Transfection was performed with 600 ng of plasmid DNA for 7 h in fresh media. After transfection, cells were incubated in fresh media or media containing gentamicin with or without various concentrations of CDX compounds for 24 h. The transfection efficiency of all constructs has been described previously [23].

### 4.4. Western Blotting

Cultured HeLa cells were washed with ice-cold PBS and lysed in RIPA buffer (50 mM Tris-HC1, pH 7.5, 400 mM NaCl, 5 mM EDTA, 1% NP-40, 0.8% sodium deoxycholate and 0.1% SDS) supplemented with protease inhibitor (Roche, Basel, Switzerland). Homogenates were left for 30 min on ice, centrifuged at 14,000 rpm for 15 min at 4 °C, and the supernatant was prepared for Western blotting using standard procedures. The total protein concentration was measured using BCA protein assay (Interchim, Montluçon, France). Western blotting of cell protein lysates was performed using Bolt Mini gels and Mini Bolt wet transfer modules (Invitrogen, Waltham, MA, USA) according to the manufacturer’s instructions. In brief, 25–30 µg protein samples were separated on 4–12% gradient precast polyacrylamide gel (Invitrogen), transferred onto a nitrocellulose membrane (Amershan™ Protran^®^, GE Healthcare, Chicago, IL, USA), and blocked with 5% non-fat milk solution for 1 h at room temperature. Membranes were incubated with specific primary antibodies overnight at 4 °C and subsequently incubated with Horseradish peroxidase-conjugated secondary antibodies (Jackson ImmunoResearch, West Grove, PA, USA). Lumi-Light, Lumi-Light Plus blotting substrate (Roche, Mannheim, Germany) and Immobilon Western Chemiluminescent HRP (Merck, Darmstadt, Germany) were used for signal detection. Blot documentation was performed with an imaging system (LAS-3000 Mini, GE Healthcare or G:Box Chemi XX6, Syngene, Schwerte, Germany). The following primary antibodies were used in the study: Anti-FLAG M2 (Sigma F1804; 1:1000, Burlington, MA, USA) and C-MeCP2 (Cell signaling CS#3456; 1:1000, Danvers, MA, USA) and Histone H3 (Abcam1791; 1:5000, Cambridge, UK). Integrated band intensities of protein bands were measured using Image J software. The full-length MeCP2 expression was normalized to the loading control Histone H3.

### 4.5. Nuclear Protein Extraction

Mouse fibroblasts were trypsinized, washed with ice-cold PBS and pelleted by centrifugation at 1000 rpm for 5 min. Cells were resuspended in cytoplasmic extract (CE) buffer (10 mM HEPES, 60 mM KCl, 1 mM EDTA, 1 mM DTT, 1 mM PMSF and 0.075% NP40 and supplemented with protease inhibitor; solution is adjusted to pH7.6), vortexed for 10 s and incubated on ice for 10 min. Lysate was then spun at 1500 rpm for 4 min. The supernatant was carefully removed and the remaining nuclear pellet was resuspended in nuclear extraction (NE) buffer (20 mM Tris HCl, 420 mM NaCl, 1.5 mM MgCl_2_, 0.2 mM EDTA, 1 mM PMSF, 25% of glycerol, and supplemented with protease inhibitor; solution is adjusted to pH 8) (proportional to half the amount of used CE-buffer). The salt concentration was adjusted by 5 M NaCl (proportional to a third of the amount of used CE buffer). Then, lysates were sonicated and incubated on ice for 30 min. Lastly, the nuclear fraction was spun at maximum speed for 10 min at 4 °C to pellet any debris. The resultant supernatants were prepared for Western blotting. 

### 4.6. Immunofluorescence

A total of 1 × 10^5^ HeLa cells were seeded and grown on coverslip. After the transfection and drug treatment, cells on coverslips were fixed in cold 4% paraformaldehyde for 20 min at RT. Immunofluorescence on transfected-HeLa cells was performed using standard procedures. Briefly, after post-fixation, cells were washed three times with cold PBS and permeabilized with 0.05 M TBS solution containing 0.5 M ammonium chloride and 0.25% Triton X-100 for 10 min. Cells were then washed with 0.05 M TBS solution, blocked with 5% normal horse serum in 0.05 M TBS for 1 h at RT and incubated with primary antibodies (Anti-FLAG M2, Sigma F1804 at 1:800 dilution; and Anti-C-MeCP2, Cell Signaling #3456 at 1:200 dilution) overnight at 4 °C. After washing, secondary antibodies (Invitrogen A11036 and A11029, 1:500 dilution) were applied for 1 h and the coverslips with cells were mounted onto the SuperFrost plus slide (Thermo Fisher Scientific, Dreieich, Germany) with proLong Gold antifade reagent with DAPI (Invitrogen Ref# P36935). The stained cells were analyzed using fluorescent microscopy with Apotome.2 (Zeiss, Jena, Germany).

### 4.7. Cell Viability Assay

Cell viability assays were conducted using Cell Counting Kit 8 (WST-8) from Abcam. HeLa cells and transfected HeLa cells were seeded in clear 96-well plates at a density of 4000 cells per well and allowed to adhere overnight. The media were the replaced with fresh media containing 800 μg/mL of gentamicin, along with either DMSO or CDX compounds. Control wells received vehicle treatment with DMSO only. Each treatment condition was performed in triplicate or quadruplicate. After 24 h, the treatment media were removed, and media containing the WST-8 reagent (10 μL reagent + 100 μL media per well) were added and incubated for 2 h at 37 °C. Blank wells contained media and the WST-8 reagent without cells. Absorbance at 460 nm was measured. The average values from triplicate wells were obtained, and the background absorbance from the blank wells was subtracted. The absorbance values from the compound-treated cells were then compared to those from the vehicle-treated cells.

### 4.8. Statistical Analysis

All immunoblots shown were representative of at least three independent replicates. Statistical analyses were performed using Prism 8 (GraphPad software). One-way ANOVA was used followed by Dunnett’s multiple comparison test. Results were considered statistically significant if *p* < 0.05. All quantitative data were presented as means (±SD.).

## Data Availability

Not applicable.

## Supplementary Materials

The following supporting information can be downloaded at: https://www.mdpi.com/article/10.3390/ijms241411665/s1.

## References

[1] Rett A. (1966). On a unusual brain atrophy syndrome in hyperammonemia in childhood. Wien. Med. Wochenschr..

[2] Hagberg B., Aicardi J., Dias K., Ramos O. (1983). A progressive syndrome of autism, dementia, ataxia, and loss of purposeful hand use in girls: Rett’s syndrome: Report of 35 cases. Ann. Neurol..

[3] Huppke P., Held M., Laccone F., Hanefeld F. (2003). The spectrum of phenotypes in females with Rett Syndrome. Brain Dev..

[4] Neul J.L., Kaufmann W.E., Glaze D.G., Christodoulou J., Clarke A.J., Bahi-Buisson N., Leonard H., Bailey M.E.S., Schanen N.C., Zappella M. (2010). Rett syndrome: Revised diagnostic criteria and nomenclature. Ann. Neurol..

[5] Amir R.E., Van den Veyver I.B., Wan M., Tran C.Q., Francke U., Zoghbi H.Y. (1999). Rett syndrome is caused by mutations in X-linked MECP2, encoding methyl-CpG-binding protein 2. Nat. Genet..

[6] Bienvenu T., Carrié A., de Roux N., Vinet M.-C., Jonveaux P., Couvert P., Villard L., Arzimanoglou A., Beldjord C., Fontes M. (2000). MECP2 mutations account for most cases of typical forms of Rett syndrome. Hum. Mol. Genet..

[7] Neul J.L., Fang P., Barrish J., Lane J., Caeg E.B., Smith E.O., Zoghbi H., Percy A., Glaze D.G. (2008). Specific mutations in methyl-CpG-binding protein 2 confer different severity in Rett syndrome. Neurology.

[8] (2022). Correction to ‘MeCP2 binds to nucleosome free (linker DNA) regions and to H3K9/H3K27 methylated nucleosomes in the brain’. Nucleic Acids Res..

[9] Lykke-Andersen S., Jensen T.H. (2015). Faculty Opinions recommendation of Nonsense-mediated mRNA decay: An intricate machinery that shapes transcriptomes. Nat. Rev. Mol. Cell Biol..

[10] Howard M., Frizzell R.A., Bedwell D.M. (1996). Aminoglycoside antibiotics restore CFTR function by overcoming premature stop mutations. Nat. Med..

[11] Dabrowski M., Bukowy-Bieryllo Z., Zietkiewicz E. (2018). Advances in therapeutic use of a drug-stimulated translational readthrough of premature termination codons. Mol. Med..

[12] Keeling K.M., Xue X., Gunn G., Bedwell D.M. (2014). Therapeutics Based on Stop Codon Readthrough. Annu. Rev. Genom. Hum. Genet..

[13] Nagel-Wolfrum K., Möller F., Penner I., Baasov T., Wolfrum U. (2016). Targeting Nonsense Mutations in Diseases with Translational Read-Through-Inducing Drugs (TRIDs). Biodrugs.

[14] Prokhorova I., Altman R.B., Djumagulov M., Shrestha J.P., Urzhumtsev A., Ferguson A., Chang C.-W.T., Yusupov M., Blanchard S.C., Yusupova G. (2017). Aminoglycoside interactions and impacts on the eukaryotic ribosome. Proc. Natl. Acad. Sci. USA.

[15] Sharma J., Keeling K.M., Rowe S.M. (2020). Pharmacological approaches for targeting cystic fibrosis nonsense mutations. Eur. J. Med. Chem..

[16] Finkel R.S. (2010). Read-Through Strategies for Suppression of Nonsense Mutations in Duchenne/Becker Muscular Dystrophy: Aminoglycosides and Ataluren (PTC124). J. Child Neurol..

[17] Malik V., Rodino-Klapac L.R., Viollet L., Mendell J.R. (2010). Aminoglycoside-induced mutation suppression (stop codon readthrough) as a therapeutic strategy for Duchenne muscular dystrophy. Ther. Adv. Neurol. Disord..

[18] Omachi K., Kai H., Roberge M., Miner J.H. (2022). NanoLuc reporters identify COL4A5 nonsense mutations susceptible to drug-induced stop codon readthrough. iScience.

[19] Has C., Sayar S.B., Zheng S., Chacón-Solano E., Condrat I., Yadav A., Roberge M., Laguzzi F.L. (2021). Read-Through for Nonsense Mutations in Type XVII Collagen-Deficient Junctional Epidermolysis Bullosa. J. Investig. Dermatol..

[20] Floquet C., Deforges J., Rousset J.-P., Bidou L. (2010). Rescue of non-sense mutated p53 tumor suppressor gene by aminoglycosides. Nucleic Acids Res..

[21] Bykov V.J.N., Eriksson S.E., Bianchi J., Wiman K.G. (2018). Targeting mutant p53 for efficient cancer therapy. Nat. Rev. Cancer.

[22] Abreu R.B.V., Gomes T.T., Nepomuceno T.C., Li X., Fuchshuber-Moraes M., De Gregoriis G., Suarez-Kurtz G., Monteiro A.N.A., Carvalho M.A. (2022). Functional Restoration of BRCA1 Nonsense Mutations by Aminoglycoside-Induced Readthrough. Front. Pharmacol..

[23] Brendel C., Klahold E., Gärtner J., Huppke P. (2009). Suppression of Nonsense Mutations in Rett Syndrome by Aminoglycoside Antibiotics. Pediatr. Res..

[24] Brendel C., Belakhov V., Werner H., Wegener E., Gärtner J., Nudelman I., Baasov T., Huppke P. (2010). Readthrough of nonsense mutations in Rett syndrome: Evaluation of novel aminoglycosides and generation of a new mouse model. J. Mol. Med..

[25] Popescu A.C., Sidorova E., Zhang G., Eubanks J.H. (2010). Aminoglycoside-mediated partial suppression of *MECP2* nonsense mutations responsible for Rett syndrome in vitro. J. Neurosci. Res..

[26] Lacy M.K., Nicolau D.P., Nightingale C.H., Quintiliani R. (1998). The Pharmacodynamics of Aminoglycosides. Clin. Infect. Dis..

[27] Nudelman I., Rebibo-Sabbah A., Cherniavsky M., Belakhov V., Hainrichson M., Chen F., Schacht J., Pilch D.S., Ben-Yosef T., Baasov T. (2009). Development of Novel Aminoglycoside (NB54) with Reduced Toxicity and Enhanced Suppression of Disease-Causing Premature Stop Mutations. J. Med. Chem..

[28] Nudelman I., Glikin D., Smolkin B., Hainrichson M., Belakhov V., Baasov T. (2010). Repairing faulty genes by aminoglycosides: Development of new derivatives of geneticin (G418) with enhanced suppression of diseases-causing nonsense mutations. Bioorg. Med. Chem..

[29] Vecsler M., Ben Zeev B., Nudelman I., Anikster Y., Simon A.J., Amariglio N., Rechavi G., Baasov T., Gak E. (2011). Ex Vivo Treatment with a Novel Synthetic Aminoglycoside NB54 in Primary Fibroblasts from Rett Syndrome Patients Suppresses MECP2 Nonsense Mutations. PLoS ONE.

[30] Bidou L., Bugaud O., Belakhov V., Baasov T., Namy O. (2017). Characterization of new-generation aminoglycoside promoting premature termination codon readthrough in cancer cells. RNA Biol..

[31] Baradaran-Heravi A., Niesser J., Balgi A.D., Choi K., Zimmerman C., South A.P., Anderson H.J., Strynadka N.C., Bally M.B., Roberge M. (2017). Gentamicin B1 is a minor gentamicin component with major nonsense mutation suppression activity. Proc. Natl. Acad. Sci. USA.

[32] Popadynec M., Baradaran-Heravi A., Alford B., Cameron S.A., Clinch K., Mason J.M., Rendle P.M., Zubkova O.V., Gan Z., Liu H. (2021). Reducing the Toxicity of Designer Aminoglycosides as Nonsense Mutation Readthrough Agents for Therapeutic Targets. ACS Med. Chem. Lett..

[33] Keeling K.M., Wang D., Dai Y., Murugesan S., Chenna B., Clark J., Belakhov V., Kandasamy J., Velu S.E., Baasov T. (2013). Attenuation of Nonsense-Mediated mRNA Decay Enhances In Vivo Nonsense Suppression. PLoS ONE.

[34] McHugh D.R., Cotton C.U., Hodges C.A. (2020). Synergy between Readthrough and Nonsense Mediated Decay Inhibition in a Murine Model of Cystic Fibrosis Nonsense Mutations. Int. J. Mol. Sci..

[35] Ferguson M.W., Gerak C., Chow C.C.T., Rastelli E.J., Elmore K.E., Stahl F., Hosseini-Farahabadi S., Baradaran-Heravi A., Coltart D.M., Roberge M. (2019). The antimalarial drug mefloquine enhances TP53 premature termination codon readthrough by aminoglycoside G418. PLoS ONE.

[36] Sharma J., Du M., Wong E., Mutyam V., Li Y., Chen J., Wangen J., Thrasher K., Fu L., Peng N. (2021). A small molecule that induces translational readthrough of CFTR nonsense mutations by eRF1 depletion. Nat. Commun..

[37] Baradaran-Heravi A., Balgi A.D., Hosseini-Farahabadi S., Choi K., Has C., Roberge M. (2021). Effect of small molecule eRF3 degraders on premature termination codon readthrough. Nucleic Acids Res..

[38] Baradaran-Heravi A., Bauer C.C., Pickles I.B., Hosseini-Farahabadi S., Balgi A.D., Choi K., Linley D.M., Beech D.J., Roberge M., Bon R.S. (2022). Nonselective TRPC channel inhibition and suppression of aminoglycoside-induced premature termination codon readthrough by the small molecule AC1903. J. Biol. Chem..

[39] Baradaran-Heravi A., Balgi A.D., Zimmerman C., Choi K., Shidmoossavee F.S., Tan J.S., Bergeaud C., Krause A., Flibotte S., Shimizu Y. (2016). Novel small molecules potentiate premature termination codon readthrough by aminoglycosides. Nucleic Acids Res..

[40] Frew J., Baradaran-Heravi A., Balgi A.D., Wu X., Yan T., Arns S., Shidmoossavee F.S., Tan J., Jaquith J.B., Jansen-West K.R. (2020). Premature termination codon readthrough upregulates progranulin expression and improves lysosomal function in preclinical models of GRN deficiency. Mol. Neurodegener..

[41] Bonetti B., Fu L., Moon J., Bedwell D.M. (1995). The Efficiency of Translation Termination is Determined by a Synergistic Interplay Between Upstream and Downstream Sequences inSaccharomyces cerevisiae. J. Mol. Biol..

[42] Cridge A.G., Crowe-McAuliffe C., Mathew S.F., Tate W.P. (2018). Eukaryotic translational termination efficiency is influenced by the 3′ nucleotides within the ribosomal mRNA channel. Nucleic Acids Res..

[43] Wangen J.R., Green R. (2020). Stop codon context influences genome-wide stimulation of termination codon readthrough by aminoglycosides. Elife.

[44] Linde L., Boelz S., Nissim-Rafinia M., Oren Y.S., Wilschanski M., Yaacov Y., Virgilis D., Neu-Yilik G., Kulozik A.E., Kerem E. (2007). Nonsense-mediated mRNA decay affects nonsense transcript levels and governs response of cystic fibrosis patients to gentamicin. J. Clin. Investig..

[45] Hinzpeter A., Aissat A., de Becdelièvre A., Bieth E., Sondo E., Martin N., Costes B., Costa C., Goossens M., Galietta L.J. (2012). Alternative Splicing of In-Frame Exon Associated with Premature Termination Codons: Implications for Readthrough Therapies. Hum. Mutat..

[46] Blanchet S., Cornu D., Argentini M., Namy O. (2014). New insights into the incorporation of natural suppressor tRNAs at stop codons in Saccharomyces cerevisiae. Nucleic Acids Res..

[47] Roy B., Leszyk J.D., Mangus D.A., Jacobson A. (2015). Nonsense suppression by near-cognate tRNAs employs alternative base pairing at codon positions 1 and 3. Proc. Natl. Acad. Sci. USA.

[48] Loenarz C., Sekirnik R., Thalhammer A., Ge W., Spivakovsky E., Mackeen M.M., McDonough M.A., Cockman M.E., Kessler B.M., Ratcliffe P.J. (2014). Hydroxylation of the eukaryotic ribosomal decoding center affects translational accuracy. Proc. Natl. Acad. Sci. USA.

[49] Beznosková P., Wagner S., Jansen M.E., von der Haar T., Valášek L.S. (2015). Translation initiation factor eIF3 promotes programmed stop codon readthrough. Nucleic Acids Res..

[50] Feng T., Yamamoto A., Wilkins S.E., Sokolova E., Yates L.A., Münzel M., Singh P., Hopkinson R.J., Fischer R., Cockman M.E. (2014). Optimal Translational Termination Requires C4 Lysyl Hydroxylation of eRF1. Mol. Cell.

[51] Zhang H., Lyu Z., Fan Y., Evans C.R., Barber K.W., Banerjee K., Igoshin O.A., Rinehart J., Ling J. (2020). Metabolic stress promotes stop-codon readthrough and phenotypic heterogeneity. Proc. Natl. Acad. Sci. USA.

[52] McCaughan K.K., Brown C.M., Dalphin M.E., Berry M.J., Tate W.P. (1995). Translational termination efficiency in mammals is influenced by the base following the stop codon. Proc. Natl. Acad. Sci. USA.

[53] Dabrowski M., Bukowy-Bieryllo Z., Zietkiewicz E. (2015). Translational readthrough potential of natural termination codons in eucaryotes—The impact of RNA sequence. RNA Biol..

[54] Floquet C., Hatin I., Rousset J.-P., Bidou L. (2012). Statistical Analysis of Readthrough Levels for Nonsense Mutations in Mammalian Cells Reveals a Major Determinant of Response to Gentamicin. PLoS Genet..

[55] Manuvakhova M., Keeling K., Bedwell D.M. (2000). Aminoglycoside antibiotics mediate context-dependent suppression of termination codons in a mammalian translation system. RNA.

[56] Howard M.T., Shirts B.H., Petros L.M., Flanigan K.M., Gesteland R.F., Atkins J.F. (2000). Sequence specificity of aminoglycoside-induced stop condon readthrough: Potential implications for treatment of Duchenne muscular dystrophy. Ann. Neurol..

[57] Hosseini-Farahabadi S., Baradaran-Heravi A., Zimmerman C., Choi K., Flibotte S., Roberge M. (2021). Small molecule Y-320 stimulates ribosome biogenesis, protein synthesis, and aminoglycoside-induced premature termination codon readthrough. PLoS Biol..

[58] Merritt J.K., Collins B.E., Erickson K.R., Dong H., Neul J.L. (2020). Pharmacological read-through of R294X Mecp2 in a novel mouse model of Rett syndrome. Hum. Mol. Genet..

[59] Pitcher M.R., Herrera J.A., Buffington S.A., Kochukov M.Y., Merritt J.K., Fisher A.R., Schanen N.C., Costa-Mattioli M., Neul J.L. (2015). Rett syndrome like phenotypes in the R255X Mecp2 mutant mouse are rescued by MECP2 transgene. Hum. Mol. Genet..

[60] Gunn G., Dai Y., Du M., Belakhov V., Kandasamy J., Schoeb T.R., Baasov T., Bedwell D.M., Keeling K.M. (2014). Long-term nonsense suppression therapy moderates MPS I-H disease progression. Mol. Genet. Metab..

[61] Crawford D.K., Alroy I., Sharpe N., Goddeeris M.M., Williams G. (2020). ELX-02 Generates Protein via Premature Stop Codon Read-Through without Inducing Native Stop Codon Read-Through Proteins. Experiment.

