# Supplementary material for: Evaluation of Novel Enhancer Compounds in Gentamicin-Mediated Readthrough of Nonsense Mutations in Rett Syndrome
Source: Int J Mol Sci. 2023 Jul 19;24(14):11665. doi: 10.3390/ijms241411665 (PMC10380790; doi:10.3390/ijms241411665)
Supplement: Supplementary file 1 [file ijms-24-11665-s001.zip › ijms-2491055-Supplementary -Revised.pdf]

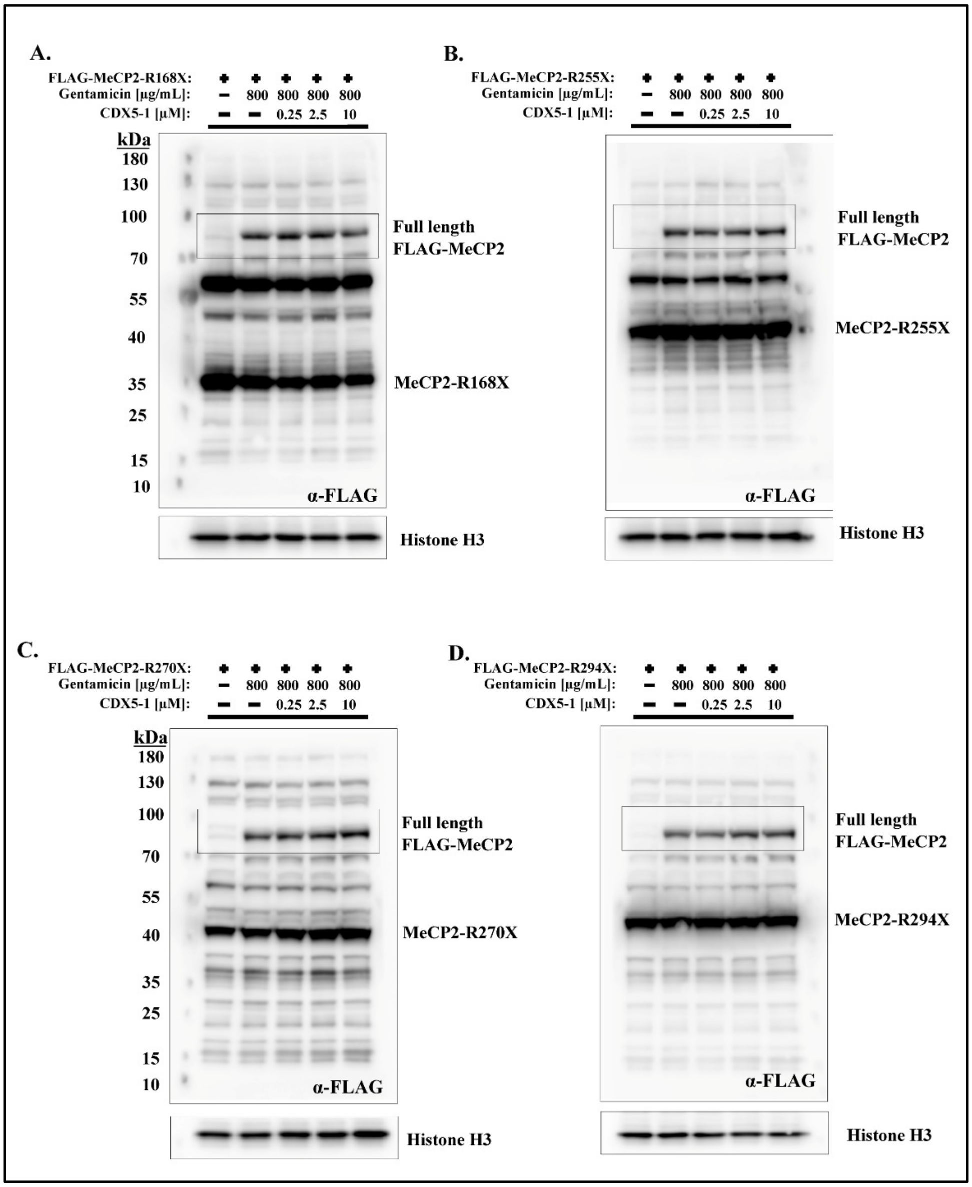

Figure S1. Analysis of potential MeCP2 truncated proteins following the co-treatment of Gentamicin and CDX5-1. **(A).** Western blot analysis of R168X, **(B).** R255X, **(C).** R270X, and **(D).** R290X transfected HeLa cells treated with 800 μg/mL gentamicin and the indicated concentrations of CDX5-1 for 24hr, and probed with the Anti-FLAG antibody. Note that MeCP2-R168X,-R255X, -R270X and -R294X are the truncated FLAG-MeCP2 isoforms.

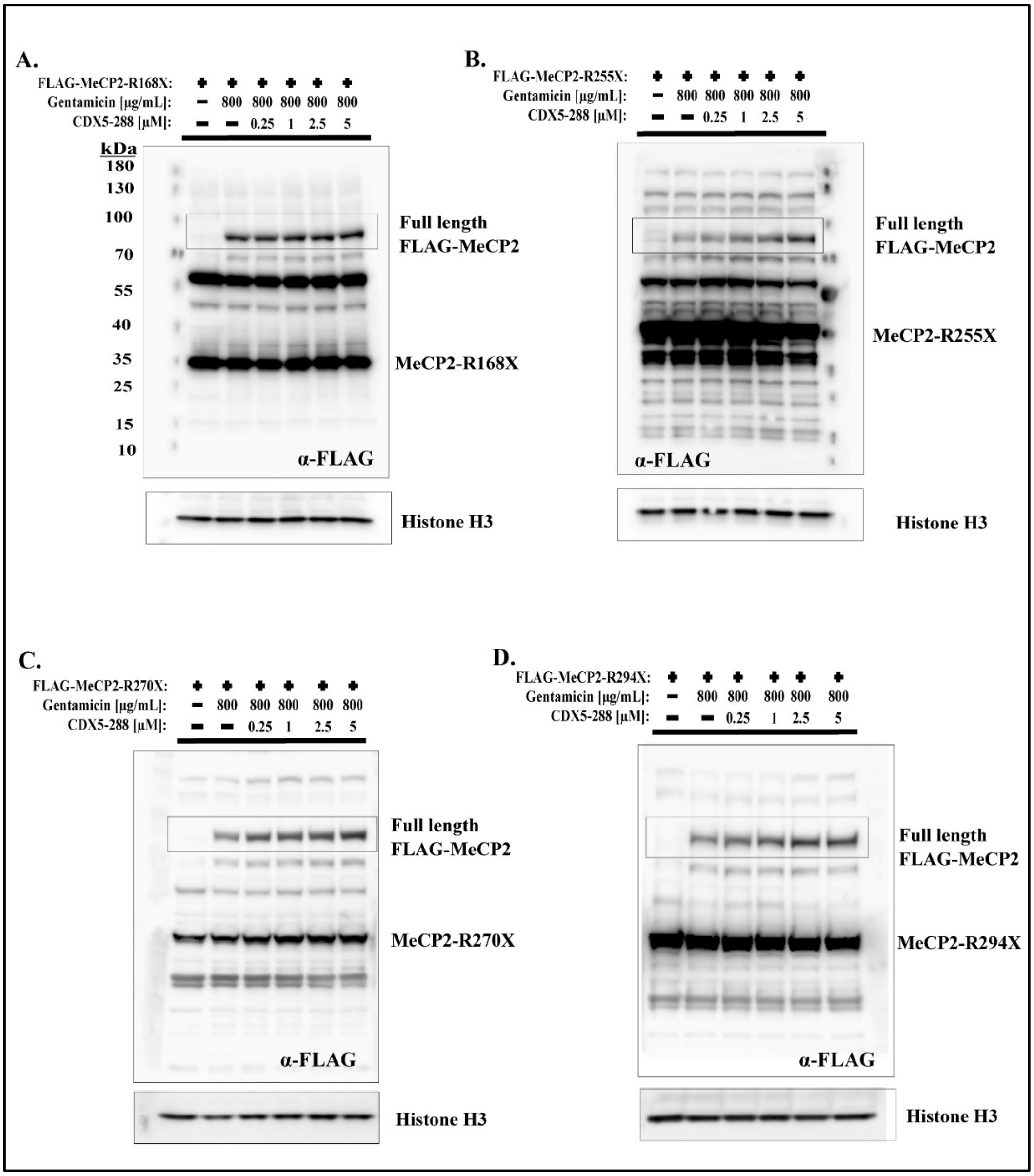

Figure S2. Analysis of potential MeCP2 truncated proteins following the co-treatment of Gentamicin and CDX5-288. **(A).** Western blot analysis of R168X, **(B).** R255X, **(C).** R270X, and **(D).** R290X transfected HeLa cells treated with 800 μg/mL gentamicin and the indicated concentrations of CDX5-288 for 24hr, and probed with the Anti-FLAG antibody. Note that MeCP2-R168X,-R255X, -R270X and -R294X are the truncated FLAG-MeCP2 isoforms.

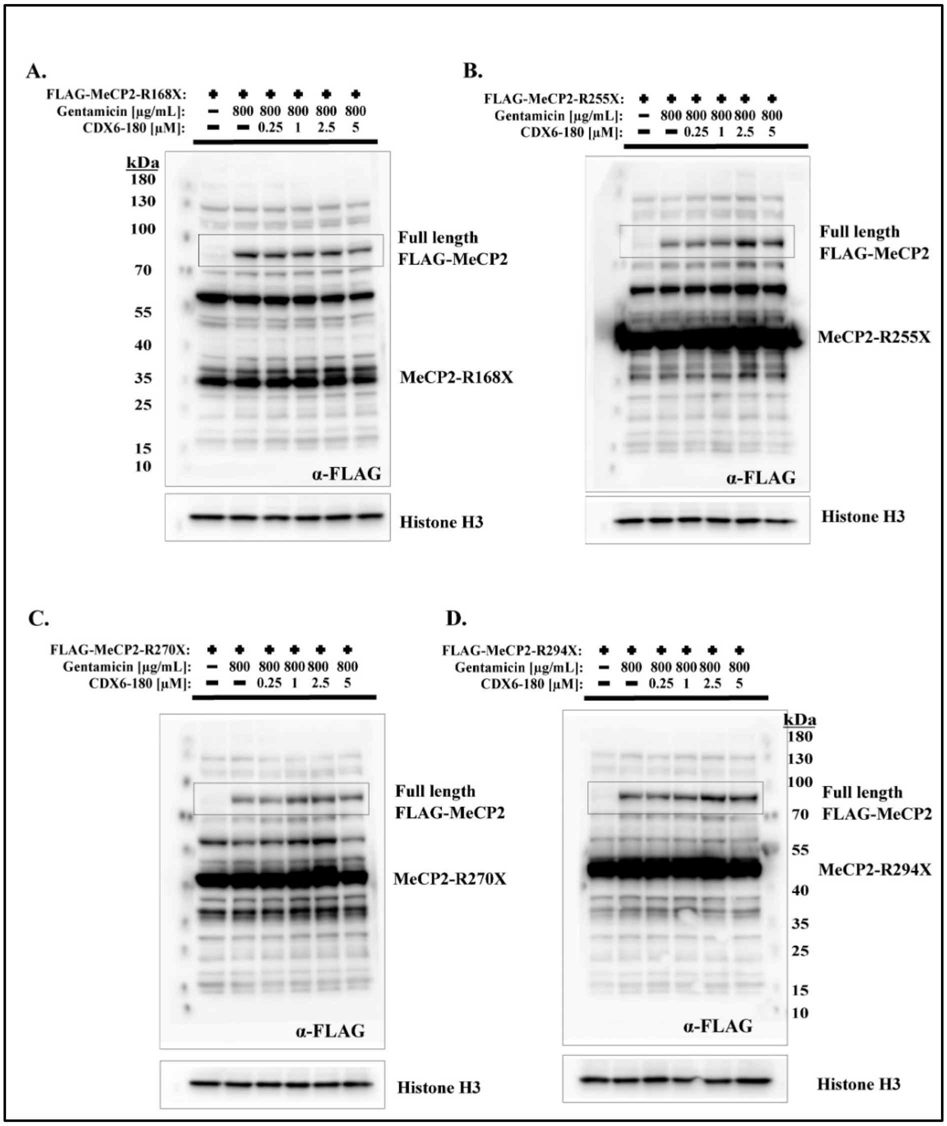

Figure S3. Analysis of potential MeCP2 truncated proteins following the co-treatment of Gentamicin and CDX6-180. (**A**). Western blot analysis of R168X, (**B**). R255X, (**C**). R270X, and (**D**). R290X transfected HeLa cells treated with 800 µg/mL gentamicin and the indicated concentrations of CDX6-180 for 24hr, and probed with the Anti-FLAG antibody. Note that MeCP2-R168X,-R255X, -R270X and -R294X are the truncated FLAG-MeCP2 isoforms.

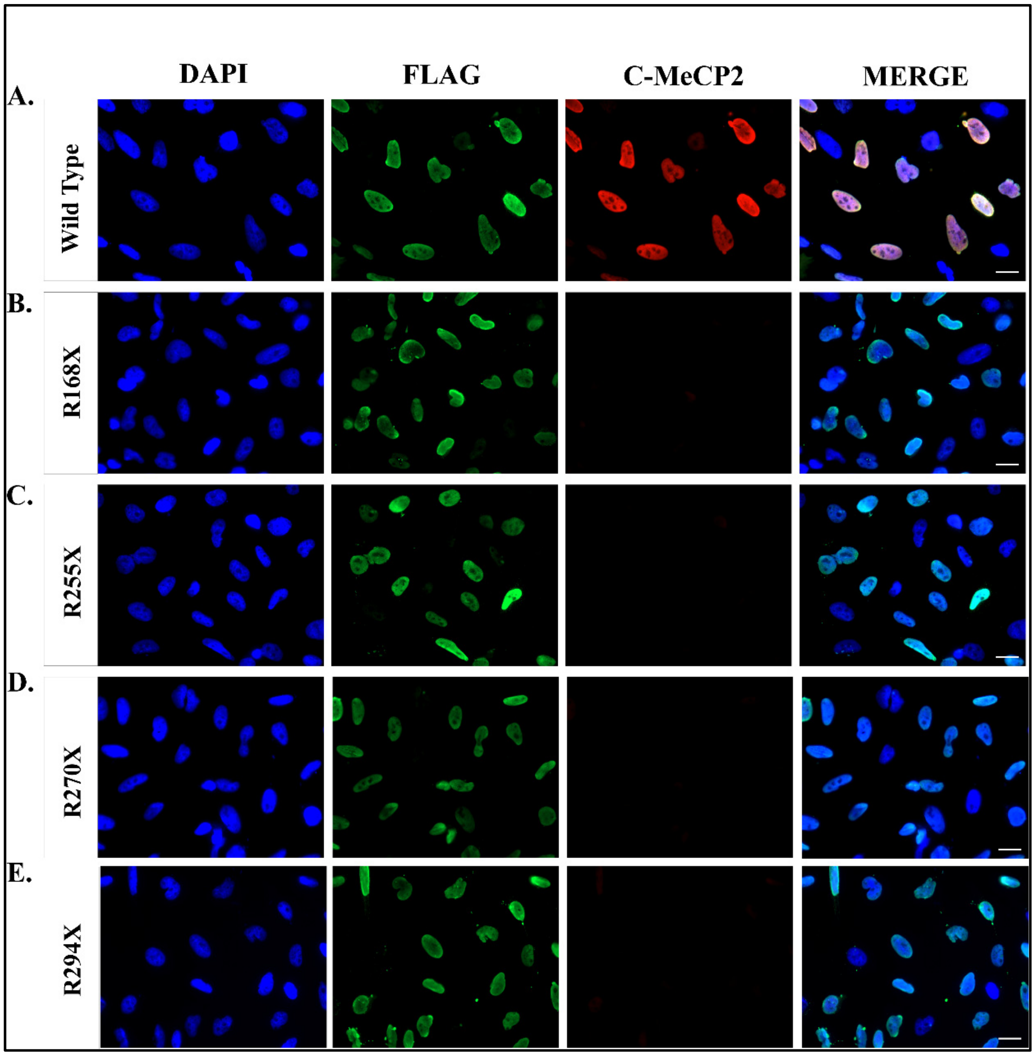

Figure S4. No signal corresponding to C-MeCP2 was detected in untreated cells expressing mutated MeCP2 isoforms. **(A).** Immunofluorescence studies of wild type-MeCP2, **(B).** R168X, **(C).** R255X, **(D).** R270X, and **(E).** R290X transfected HeLa cells without any treatment. Scale bars: 20 μm.
